# Macrophages, Metabolites, and Nucleosomes: Chromatin at the Intersection between Aging and Inflammation

**DOI:** 10.3390/ijms221910274

**Published:** 2021-09-24

**Authors:** Michael C. Church, Jerry L. Workman, Tamaki Suganuma

**Affiliations:** Stowers Institute for Medical Research, 1000 E. 50th Street, Kansas City, MO 64110, USA; MChurch@stowers.org (M.C.C.); jlw@stowers.org (J.L.W.)

**Keywords:** pro-inflammation, chronic inflammation, chromatin, metabolism, aging, histone modifications, inflammaging, transcription

## Abstract

Inflammation is the body’s means of defense against harmful stimuli, with the ultimate aim being to restore homeostasis. Controlled acute inflammation transiently activates an immune response and can be beneficial as protection against infection or injury. However, dysregulated inflammatory responses, including chronic inflammation, disrupt the immune system’s ability to maintain homeostatic balance, leading to increased susceptibility to infection, continuous tissue damage, and dysfunction. Aging is a risk factor for chronic inflammation; their coincidence is termed “inflammaging”. Metabolic disorders including obesity, neurodegenerative diseases, and atherosclerosis are often encountered in old age. Therefore, it is important to understand the mechanistic relationship between aging, chronic inflammation, and metabolism. It has been established that the expression of inflammatory mediators is transcriptionally and translationally regulated. In addition, the post-translational modification of the mediators plays a crucial role in the response to inflammatory signaling. Chromatin regulation responds to metabolic status and controls homeostasis. However, chromatin structure is also changed by aging. In this review, we discuss the functional contributions of chromatin regulation to inflammaging.

## 1. Introduction 

Aged individuals are susceptible to many chronic diseases that are less common in youth, and in addition to the mental and physical burdens that this places on an individual and their immediate family, the long-term treatment of chronic conditions may become prohibitively expensive for societies. Chronic inflammation is a common feature of human aging, and this can lead to infirmity as cells and organs lose the capacity to recover with age [1]. Thus, as medical science improves lifespans, the proportion of unhealthy individuals may paradoxically increase. It is obvious that strategies to impede or even to reverse the effects of aging are of great urgency.

The purpose of a successful inflammatory response is to eliminate or isolate an infectious agent or heal sterile tissue damage, and subsequently to initiate the process of recovery. Acute inflammation is a short-term process, and ideally it is resolved with minimal lasting tissue damage. The classic signs of acute inflammation are redness, heat, pain, swelling and loss of function [2]. Acute inflammation in the absence of infection can also promote wound regeneration or repair, depending on the severity of the tissue damage [3]. In contrast, the chronic, sterile inflammation that results from repeated immune stimulation over time may be the result of the degeneration of a number of receptors that activate the innate immune system in elderly individuals [1]. Adaptive immunity is also impaired in the elderly, including perturbations in T cell populations (decreased naïve and increased memory T cells), and the accumulation of atypical B cells, can lead to reduced immune function and autoimmune disease [4,5]. A “generic” inflammatory pathway includes Inducers, Sensors, Mediators and Effectors [6]. An example of this pathway in action would be the stimulation of Sensors, such as the Toll-Like Receptors (TLRs) present on macrophages or mast cells by a microbe (Inducer), leading to the production of cytokines (Mediator), which act on target tissues (Effectors) in order to promote the recruitment of pathogen-destroying cells to the affected area [2]. These actions lead to the signs of inflammation through vasodilation, edema, and the presence of pain-promoting prostaglandins in the affected tissue [7]. The resolution of inflammation occurs when the injury/infection is resolved and the inflammation-promoting effectors are replaced by pro-resolution mediators, such as anti-inflammatory cytokines (e.g., IL-10 & TGFβ) and lipoxins, beginning the process of repair [2,8]. If the infection or injury is not resolved, then chronic inflammation can ensue. Persistent infections, such as with *Mycobacterium tuberculosis*, can lead to unresolved, chronic inflammation that causes lung tissue damage and not only results in failure to clear the infection, but promotes transmission through generation of infectious aerosols [9]. In addition to unresolved infection or tissue damage, chronic inflammation can be the result of diseases that are less easily explained. Several instances of chronic inflammation are correlated with diseases that occur in aged individuals, including obesity, diabetes, atherosclerosis, and Alzheimer’s disease [10,11,12].

Low-grade inflammation is often observed as part of aging. This phenomenon has been termed “inflammaging” [13]. In addition to a general decline in function during aging, the nature of the immune system also changes, in a phenomenon known as immunosenescence [14]. This accounts for the reduced ability of the elderly to respond to antigens and correlates with increased susceptibility to infections. It has been known for some time that cellular nutrition can influence inflammatory responses, and the term “metaflammation” has been employed to describe the metabolic basis for this [1]. High nutrient intake fuels chronic inflammation, and dietary restriction may improve markers of inflammation [15]. Even at the most fundamental level, the production of the vast array of molecules required to regulate inflammation uses a wide assortment of compounds derived from metabolites, such as the enzymatic digestion of cell membrane phospholipids to produce prostaglandins, or the use of S-adenosyl methionine (SAMe)/adenosine triphosphate (ATP) as a cofactor to influence cytokine production at the transcriptional level [16,17]. 

The co-ordination of the many processes that contribute to the effective control of the inflammatory response relating to aging is complicated, and the revelation of the mechanisms underlying this control has only recently begun. It has been found that the production of the correct inflammatory mediator in a timely manner requires exquisite control at the transcriptional level. Importantly, all eukaryotic transcription takes place in the context of the nucleoprotein complex known as chromatin [18]. In this review, we aim to emphasize the roles of chromatin regulation at the intersection between inflammation, aging, and metabolism to deepen our mechanistic understanding of inflammaging while we discuss the possibility of obtaining control over inflammaging and directions for further studies. 

## 2. Role of Chromatin Modifications in Metabolism and Aging

**Figure 1:**
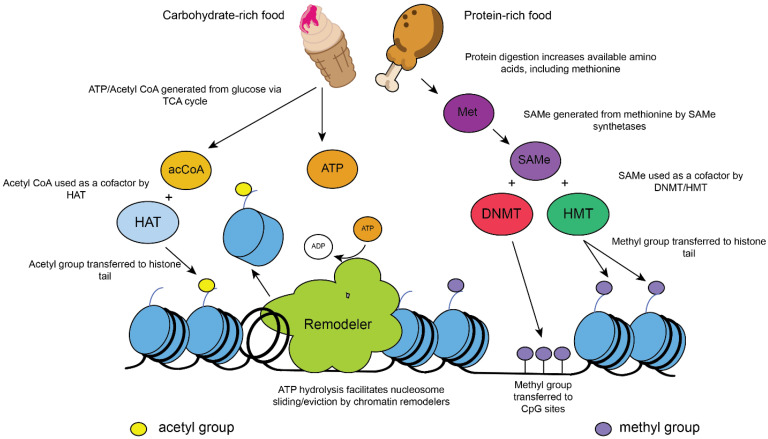
Nutrition and metabolism are directly linked to chromatin modification. (**Left**) Eating glucose-rich foods can lead to increased levels of intracellular acetyl coenzyme A (acCoA) and adenosine triphosphate (ATP) via the (tricarboxylic acid) TCA cycle. Acetyl-CoA is required as a cofactor in acetylation reactions within cells, which are catalyzed by acetyltransferases. Histone acetyltransferases (HATs) acetylate histone N-terminal tails. Acetylation is a modification associated with active gene transcription when acetylated histones are present in gene promoters/enhancers. ATP is required by ATP-dependent chromatin remodelers, which promote active transcription by sliding/evicting nucleosomes and facilitating binding sites. Alternatively, chromatin remodelers may act to produce ordered nucleosomal arrays that inhibit transcription. (**Right**) Protein-rich foods contain abundant methionine (Met), which is required for S-adenosyl methionine (SAMe) synthesis. SAMe is used as a cofactor in methylation reactions. DNA methylation is carried out by DNA methyltransferases (DNMTs), which methylate GC-rich CpG islands, commonly found in gene promoters. Methylated DNA is associated with gene silencing in mammalian cells. The methylation of N-terminal histone tails is carried out by histone methyltransferases (HMTs). The methylation of histones has differing effects on gene expression, depending on the residue involved. Tri-methylated H3K4 (H3K4me3) at 5′ open reading frames is associated with active transcription, whereas trimethylated H3K27 or H3K9 is associated with gene repression and/or silenced chromatin [23,24,25].

The basic repeating unit of chromatin is the nucleosome. Nucleosomes are histone octamers wrapped in ~146 base pairs (bp) of DNA and can occlude regulatory DNA sequences, presenting a barrier to transcription, but this can be overcome by the modification or remodeling of nucleosomes [19,20]. The histone proteins present in nucleosomes contain flexible, N-terminal “tails” that may be modified by the addition of a number of functional groups or post-translational modifications (PTMs) to their constituent amino acids. These functional groups include numerous types of acyl (including acetyl), methyl, ubiquitin, SUMO, and phosphoryl moieties, among others [18,21,22]. Since PTMs require metabolites, histone modification has been directly linked to cellular nutrition in a wide range of species [23,24,25] (Figure 1). In many cases, histone PTMs are deposited by multisubunit complexes (chromatin modifiers) that interact with transcription factors at target sites, with acetyl groups from acetyl coenzyme A (acetyl-CoA) deposited by histone acetyltransferases (HATs) and methyl groups (SAMe-derived) by histone methyltransferases (HMTs) [26] (Figure 1). Histone deacetylases (HDACs) remove the activating acetyl mark from histones, and these reversible reactions further enable “cross talk” between different post-translational modifications in gene regulation. Acetylated histone H3 is recognized by the SWI/SNF chromatin remodeling complex, which contains a bromodomain capable of recognizing/binding acetylated proteins [27]. SWI/SNF is preferentially recruited to acetylated histones at promoters/enhancers, where it slides/evicts nucleosomes in an ATP-dependent manner. SWI/SNF therefore regulates transcription by remodeling chromatin and promoting a more open chromatin configuration at these regions in yeast and mammals [27,28,29] (Figure 1). Studies of both yeast and mammalian cells have shown that SWI/SNF is required for the activation of nutrient-responsive genes, and disruption of the SWI/SNF complex impairs the cell’s ability to adapt to its environment [30,31,32] (Figure 1).

Lower nucleosome density and “fuzzy” nucleosome positioning are typically observed in aged chromatin, whereas more youthful chromatin contains well-ordered nucleosomal arrays [33]. Moreover, total histone levels change over time and are reduced in older individuals, in a phenomenon that has been observed in many organisms [34,35]. The lifespan of yeast has been studied based on replicative lifespan (RLS, measuring the maximum number of mitotic divisions a cell can sustain) and chronological lifespan (CLS, measuring the period of time a cell survives in a postmitotic state). In yeast, the deletion of the *SAS2* histone H4 lysine 16 (H4K16) acetyltransferase extended RLS [36], and reduced trimethylation of histone H3 at lysine 36 (H3K36me3) over open reading frames led to shortened RLS [37]. Moreover, strains lacking some Set1 complex/COMPASS (H3K4 methyltransferase complex conserved from yeast to human) subunits had reduced replicative lifespans [38]. H3K4me3 was required for the activation of many genes during aging, with the under-expression of de novo NAD^+^ biosynthesis genes in mutants unable to trimethylate H3K4 being of particular interest in one study [39]. However, while global histone H3 levels fall in aging yeast, the levels of H3K4me3 remain relatively stable, with the consequence that gene promoter and rDNA H3K4me3 levels rise in aged cells [39]. This may result in a loss of rDNA heterochromatin and increased insidious transcription genome-wide. Earlier work in *Caenorhabditis elegans* showed that the loss of H3K4me3 increased longevity, again implying that the link between this mark and lifespan may be complicated and could be context/organism/tissue-specific [40,41]. Work on murine hematopoietic stem cells (HSCs) demonstrated that H3K4me3 peaks increased in size in older animals and suggested that this increased methylation could contribute to HSC dysfunction in aged individuals [41].

A recent study of HSC-enriched samples from human donors determined that in older individuals (aged 65–75 years), the activating H3K27ac mark was lost from gene enhancers known to be involved in lymphoid and immune signaling when compared to H3K27ac levels in younger individuals (aged 18–30 years), suggesting that this may contribute to immunosenescence in humans [42]. In this study, some of the genes displaying a loss of enhancer H3K27ac included *ETV6* and *GFI1*, which have been demonstrated to act as tumor suppressors [10,43]. This observation ties the alteration of this histone PTM over time to increased susceptibility to cancer, another disease closely correlated with old age.

**Figure 2:**
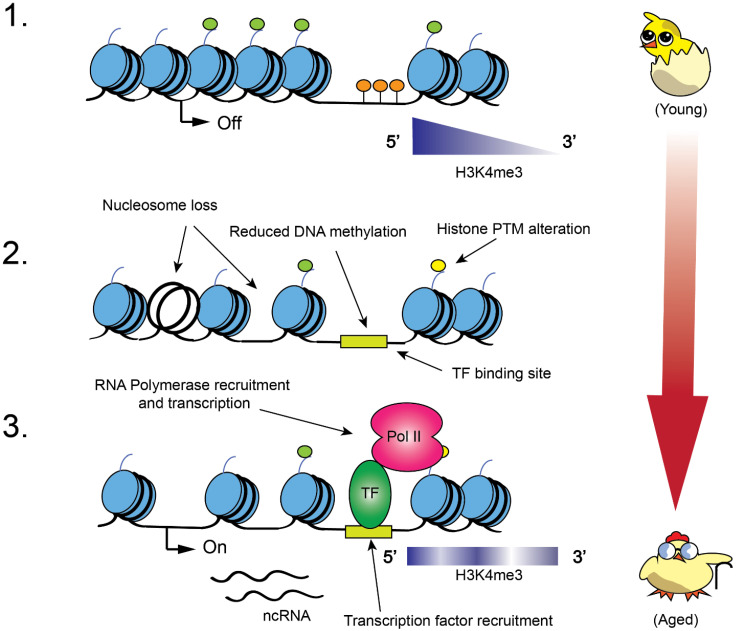
Chromatin is changed during aging. (**1**) Younger cells contain chromatin with ordered nucleosome arrays. This includes heterochromatin, which contains methylated histone H3K9 (light green circles), which in turn suppresses transcription (including spurious transcription (OFF)). Other histone marks, such as H3K4me3, are distributed appropriately in a context-dependent manner (purple wedge indicating the 5′-3′ distribution). The DNA methylation (orange circles) of CpG islands also silences genes and prevents inappropriate transcriptional activation. (**2**) As organisms age, total nucleosome numbers become reduced. This opens up regulatory elements and transcription factor (TF) binding sites (green box) on newly exposed DNA. Histone modification patterns/remodeling are also altered. (**3**) On aged chromatin, accessible chromatin may be bound inappropriately by transcription factors, which may recruit RNA Polymerase II (Pol II) and aid in the initiation of transcription. DNA methylation and heterochromatin levels are also reduced, contributing to the pervasive transcription (ON) and deterioration of cellular function. Histone modification patterns are perturbed in aged cells, which may impact longevity.

In *C. elegans*, in addition to the well-known H3K4me3 enrichment at 5′ open reading frames (ORFs), H3K4me3 enrichment in gene bodies was found to be acquired during the aging process, with the genes involved in fatty acid metabolism found to be overrepresented for this dynamic methylation, although the consequences of this were not entirely clear [44]. Therefore, not only are the absolute levels of histone modifications changed over time, but the alteration of their distribution with age may impact cellular function, underscoring the requirement for precise PTM deposition. Some of these changes are summarized in Figure 2.

Along with histone modification pattern changes, transcription factor binding patterns have been shown to be altered in aging in numerous species, including *C. elegans* and human cells [45]. FOXO/DAF-16 is one example of a well-studied transcription factor that binds open chromatin and regulates genes that promote stress resistance [45]. FOXO/DAF-16 is associated with longevity, with certain variant alleles being found in centenarians, suggesting that changes to its regulation of chromatin status prolong lifespan. Perhaps, reduced nucleosome occupancy and altered patterns of nucleosome modification in aged cells offer more opportunities for transcription factor binding at novel sites, leading to the potential for the recruitment of more chromatin modifiers and unwanted transcription events.

DNA methylation is another modification that can influence gene transcription, which has shown to be altered in aging and disease, and has been shown to be important in the function of immune cells [10,41,46,47]. DNA methylation is an inheritable modification carried out by DNA methyltransferases (DNMTs). Cytosine methylation (5-methylcytosine/5mC) has been well studied in mammals and shown to be associated with the long-term repression of gene transcription [47]. In mammals, methylation of CpG dinucleotides may occur in GC-rich “CpG islands”, which are commonly found at gene promoters. The methylation of these CpG islands may repress gene transcription by inhibiting transcription factor binding, preventing histone modifications that activate transcription, or by promoting the binding of transcriptional repressors [48]. The loss of DNA methylation of the *Cox-2* gene promoter has been shown to affect macrophage function and promote inflammation in human/murine model studies (discussed below) [16].

As described above, the deposition of “activating” histone marks can lead to nucleosome remodeling and the subsequent transcription factor binding of open chromatin. These modifications and remodeling events consume metabolites, such as SAMe and ATP, altering the cellular pool of these cofactors. SAMe is also used as a cofactor in DNA methylation. Hence, the utilization of cofactors by chromatin modifiers may contribute to energy homeostasis in addition to the regulation of metabolic gene expression. The dysfunction of chromatin modifiers alters the consumption of metabolites by chromatin and leads to an imbalance between metabolism and energy demands, as well as influencing cellular responses to aging.

## 3. Control of Inflammation Depends on Metabolic Reprogramming of Chromatin in Response to Stimuli

The balance of pro- and anti-inflammatory signals is important for maintaining health; individuals with longer lifespans have been shown to have elevated levels of anti-inflammatory molecules [49]. The macrophage is an attractive model to study, as both tissue-resident and monocyte-derived macrophages play a central role in the regulation of homeostasis and infection- and tissue damage-related inflammation, respectively [3,10]. Macrophage phenotypic differences are known to be correlated with metabolic differences between cells [50]. Traditionally, macrophages have been divided into two phenotypes: those stimulated by signals such as lipopolysaccharides (LPS) and interferon gamma (IFNγ) (M1) and those stimulated by signals such as IL-4 and IL-13 (M2). The M1 macrophages are associated with pro-inflammatory activity, whereas the M2 phenotype is associated with immune modulation/anti-inflammatory/wound healing functions, although these designations have begun to seem overly simplistic as we have learned more about different phenotypes, with macrophages appearing more varied in vivo [10,51]. The M2 macrophages are further subdivided into groups based on their inducers and perform unique roles in immune modulation and healing. These include M2a, stimulated by IL-4/13 (associated with wound healing); immunocomplex (antibody-antigen complex) and TLR agonist-stimulated M2b (known as regulatory macrophages); M2c, which is stimulated by IL-10, TGF-β or glucocorticoids (associated with anti-inflammation and phagocytosis of apoptotic cells); and M2d, stimulated by TLR and adenosine A_2A_ receptor agonists (known as tumor-associated macrophages) [52,53].

**Figure 3:**
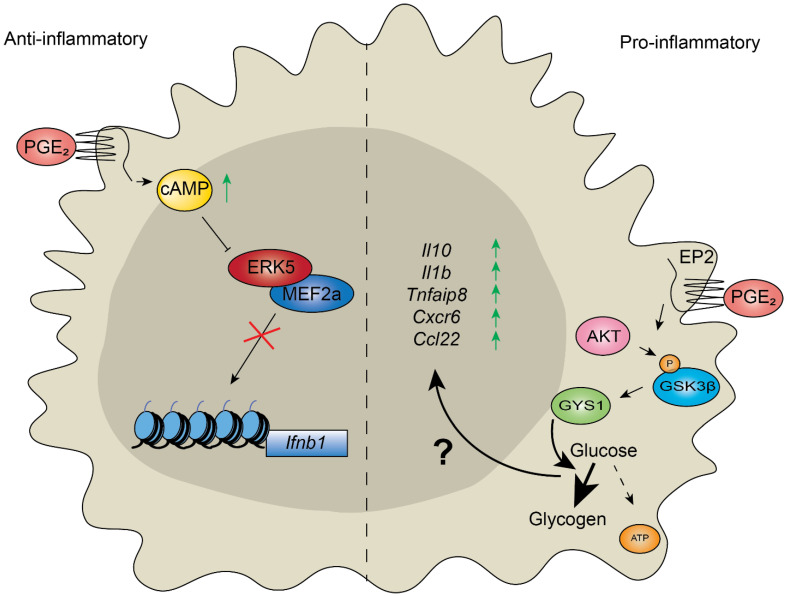
Prostaglandin E_2_ has context-dependent effects on inflammatory response in macrophages. (Left) Modulation of inflammation in human- and mouse-derived macrophages by Prostaglandin E_2_ (PGE_2_) stimulates increased cyclic adenosine monophosphate (cAMP) accumulation [55]. The transcription factor Myocyte Enhancer Factor 2A (MEF2a) activates the transcription of many lipopolysaccharide (LPS)-inducible genes in macrophages, including pro-inflammatory genes. The treatment of LPS-stimulated macrophages with PGE_2_ triggers the accumulation of cAMP, which prevents Extracellular signal-regulated kinase 5 (ERK5)-activated MEF2a recruitment to pro-inflammatory genes and therefore suppresses an inflammatory response. (Right) PGE_2_ promotes inflammation in aged microglia [60]. Increased levels of PGE_2_ in aged cells are sensed by the Prostaglandin E₂ receptor 2 (EP2), which leads to AKT-mediated phosphorylation of glycogen synthase kinase 3 β (GSK3β) at serine 9, leading to its inactivation. GSK3β is a negative regulator of glycogen synthase (GYS1), which promotes glycogen accumulation from glucose, reducing the levels of glucose-derived metabolites entering the TCA cycle (glucose flux, dashed arrow). Reduced glucose flux in aged macrophages promotes the transcription of pro-inflammatory genes by an unknown mechanism (“?”). Inhibition of the EP2 receptor, or GYS1, restores macrophage phenotypes to a more youthful state. ↑ indicates increased cAMP/ gene expression, × indicates inhibition.

Lipopolysaccharides induce the production of both pro-inflammatory and antimicrobial molecules. One study illustrates how macrophages use gene regulatory mechanisms to respond to bacterial LPS. Extended exposure to LPS leads to a reduction in pro-inflammatory transcription (“LPS tolerance”). However, in mice, it was found that genes encoding antimicrobial products could be activated in LPS-tolerant macrophages due to the differential histone modifications in antimicrobial genes and subsequent chromatin remodeling, compared to those encoding pro-inflammatory factors [54]. As both gene sets responded to the same signal from the same receptor (TLR4) in different ways, the authors determined that in the initial round of transcription following LPS stimulation, unknown transcripts were generated that would suppress pro-inflammatory transcripts in tolerant macrophages. A more recent study showed epigenetic control over the modulation of cytokine production by the prostaglandin E2 (PGE_2_) in human and murine macrophages [55]. Like other prostaglandins, PGE_2_ is a lipid compound that is generated from arachidonic acid (AA), which is cleaved from membrane phospholipids by phospholipase A2 (PLA_2_) enzymes. Arachidonic acid is utilized as a substrate by one of two cyclooxygenases (COX-1 and COX-2), yielding biological precursor prostaglandin H2 (PGH_2_). Subsequently, PGE_2_ is enzymatically produced using PGE_2_ synthase (PGES) [56,57]. Active PGE_2_ readily signals through seven prostaglandin E receptors/EPs [58]. Signaling mediated by EP2 and EP4 activates adenylate cyclase, increasing cyclic AMP (cAMP) levels [58] (Figure 3, left). Work on human and murine macrophages has shown that the Cytosolic Phospholipase A_2_ (*cPLA_2_*) and *Cox-2* genes are activated in diabetic cells, and that the activation of these genes is dependent on H3K4me3 and the loss of DNA methylation at their respective promoters in a murine wound-healing model [16]. cPLA_2_ acts at the first agonist-induced step in this pathway and was shown to require activation by the transcription factor Elk-1 and the p300 histone acetyltransferase in response to TNFα stimulation in human lung epithelial cells [57]. The cyclic AMP response element-binding protein (CBP)/p300 binds to the cAMP-response element [59]. Therefore, cAMP stimulated by EP2/EP4 in response to PGE_2_ likely selects the chromatin modifiers and target elements. The MEF2a transcription factor was shown to bind open enhancers/promoters upstream of cytokine genes (e.g., *Ifnb1*), leading to their activation upon LPS treatment (Figure 3, left). However, PGE_2_ increases cAMP levels, which prevents activation of the MEF2a by the MAP kinase (MAPK) ERK5 (Figure 3, left). Therefore, PGE_2_ modulated macrophage response to LPS by suppressing the transcription of proinflammatory cytokines (Figure 3, left).

A recent study clearly illustrates the link between nutrition, metabolism, the regulation of chromatin and inflammatory disease. Over-nutrition has been shown to be a risk factor for many inflammatory diseases and a key component in inflammaging, with a high amino acid diet being particularly pro-inflammatory [1]. Atherosclerosis is a hardening of the arteries, resulting from inflammation, that has been associated with numerous diseases, including dementia in humans [61]. It was shown that *ApoE^−/−^* mice fed a high methionine diet had a greater incidence of arterial plaques characteristic of atherosclerosis [11]. Apolipoprotein E (ApoE) is a lipoprotein involved in the catabolism of triglcyeride-rich lipoproteins, and *ApoE^−/−^* mice were used to better detect plaque formation as ApoE is known to suppress atherosclerosis [62]. Serum homocysteine (Hcy) levels, as well as levels of the pro-inflammatory cytokines IL-1β, IL-6 and TNF-α, were shown to be elevated on a high-methionine diet, and macrophages cultured in the presence of 100 μM Hcy also produced high levels of these cytokines. Interestingly, the expression levels of the SWI/SNF complex subunit SNF5/INI1 were also found to be elevated in Hcy-supplied macrophages, and were required for the expression of IL-1β. IL-1β activation requires the monomethylation of lysine 4 of histone H3 (H3K4me1), and this occurred in a SNF5-dependent manner, as SNF5 was necessary for the inhibition of the demethylase KDM1A, which removed H3K4me1 [11]. 

The differentiation of monocytes into macrophages is strongly associated with the epigenetic state of the cell, with SWI/SNF and loss of DNA methylation essential for this process [63,64]. In one example, 114 genomic regions were found to be differentially methylated between human monocytes and macrophages, with the demethylation of these sites being key to the activation of macrophage-specific genes (involved in actin cytoskeleton, phagocytosis and innate immunity) and subsequent differentiation [63]. These studies demonstrate that the modification of chromatin and transcription factors by post-translational modification is paramount in both pro-inflammatory and homeostatic activities in immune cells.

## 4. Chromatin Modifiers Regulate Aging and Chronic Inflammatory Response 

Cellular metabolism needs to change to meet the nutritional and other homeostatic demands of the cell during aging [33]. For instance, hydrogen sulfide, which is a product of sulfur metabolism, has been proposed as essential for the benefits of dietary restriction on lifespan extension in yeast and animal models [65]. In human cells, the disruption of sulfur amino acid catabolism increases reactive oxygen species, leading to amyloid beta accumulation, a typical phenotype of Alzheimer’s disease [66]. 

Macrophages resident in the brain are known as microglia, which are embryo-derived myeloid cells whose functions largely involve the clearance of debris [10]. Thus, with age, the ability of these microglia to dispose of misfolded proteins is impaired, and this impairment is associated with Alzheimer’s disease [12]. While younger human macrophages could use glucose, pyruvate, lactate and glutamine as carbon sources, aged macrophages could only use glucose, increasing their susceptibility to a pro-inflammatory phenotype [60]. During aging, levels of PGE_2_ increase, and this has been linked to disease, including neurodegenerative diseases [67,68]. Notably, it was found that increased PGE_2_ levels suppressed glucose flux in aged microglia in a manner mediated by the EP2 [60] (the detailed mechanism is illustrated in Figure 3, right). Limiting glucose flux was found to be responsible for the shift to a more pro-inflammatory macrophage polarization, and the inhibition of the EP2 restored cells to a younger phenotype, and even reversed cognitive decline in mice [60]. As discussed earlier, PGE_2_-treated macrophages can reduce the production of pro-inflammatory effectors (IL-1β, IL-6, and TNF-α) through a program of chromatin remodeling in response to intracellular signals [69,70]. In healthy individuals, this prevents chronic inflammation as part of normal homeostasis, but obesity may interrupt the reduction of pro-inflammation, as seen in the promotion of tumor growth by excess PGE_2_ in obesity-related cancer [71]. Together, metabolic disorders readily tilt the regulation of homeostasis with aging towards inflammation. Chromatin modifiers respond to cellular metabolism by sensing available cofactors and simultaneously regulate gene expression in response to inflammatory stimuli. However, aging leads to a loosening of the structure of chromatin (described above). Therefore, the macrophage response to PGE_2_, which is regulated at the level of chromatin modification, may be blunted by aging. 

## 5. Chromatin Modifiers Associated with RNA Synthesis and Metabolic Sinks May Contribute to Inflammaging 

In addition to affecting gene transcription, the catalytic activities of chromatin modifiers also regulate RNA synthesis and translation. The removal of histone H3K4me2 demethylase activity of the LSD1/KDM1A histone demethylase in MCM7 human breast cancer cells reduces the expression of the RNA-induced silencing complex, leading to the increased transcription of endogenous retroviral elements and dsRNA synthesis. These dsRNAs are recognized by pattern recognition receptors, including interferon-induced-with-helicase C domain 1 (IFIH1, encoding MDM5) [72]. LSD1 deplation activates the IFN pathway and stimulates immunogenicity, as observed in a LSD1 knockout mouse syngeneic tumor model [72]. Hence, a loss of LSD1 activity, which requires flavin adenine dinucleotide (FAD) synthesized from the vitamin riboflavin and ATP [73], can induce receptors of innate immune system that recognize DNA and RNA [74]. It remains unclear whether FAD levels are increased in cells bearing chronic inflammation. 

The metabolic pathways associated with IFN may also play important roles in the tolerance of viral infection. Moreover, insights into the differences between acute metabolic responses to bacteria and viruses may be valuable to the understanding tissue damage tolerance. The intake of glucose-inhibiting ketogenesis prevents neuronal adaptation to bacterial inflammation [75]. However, glucose utilization is essential for neuronal adaptation to viral infection as injections of poly (I:C) (analogue of virus immunostimulant) and 2-deoxy-D-glucose (2DG) (blockade of glucose utilization) cause lethality to wild-type mice but not to IFNα-receptor deficient mice [75]. 

Interferon gamma (IFNγ) has been shown to activate and phosphorylate double-stranded RNA-dependent protein kinase R (PKR)/EIF2AK2 in response to dsRNA in order to regulate the translation yield of IFNγ itself [76]. In mammalian cells, PKR activates JNK and insulin receptor substrate 1 and inhibits eIF2*α* in response to palmitic acid or thapsigargin, which stimulate obesity, resulting in the inhibition of insulin signaling [77]. The loss of PKR prevents lipid-induced insulin resistance in mice [77]. Therefore, translation is suppressed while PKR is activated by obesity or viral pathogens. In mammals and *Drosophila*, innate PKR activity is suppressed by the association of the Ada Two-A-containing (ATAC) acetyltransferase complex and molybdopterin (MPT) synthase, leading to the suppression of JNK and the promotion of translation [78]. The activation of translation may prevent cytokine production from changing from an acute response into chronic inflammation in response to viral infections.

Histone modifications unrelated to transcription may contribute to the control of inflammation. The supplementation of SAMe to lipopolysaccharide (LPS)-stimulated human bone marrow-derived macrophages induces the expression of IL-1β but not of TNF-α [79]. The addition of LPS to serine-starved macrophages increases the methylation levels of H3K36me3 and H3K4me3 (active transcription marks) but not of H3K27me3 (suppressive transcription mark) [79]. Thus, it has been suggested that LPS-induced inflammation is driven by de novo synthesized SAMe, which may promote transcription simultaneously [79]. However, in yeast, core histone H3K36me3 and H3K4me3 function as a methyl-sink when phospholipid methylation is lost [24]. Sulfur amino acid catabolism and fatty acid beta oxidation requiring MPT-synthase-associating complex (MPTAC) maintains SAMe levels in human cells [66]. Therefore, the disruption of lipid metabolism and the loss of basal SAMe levels may also trigger pro-inflammation induced by LPS-stimulation. To understand the causes of inflammaging it is important to study whether SAMe metabolism, which is connected to lipid metabolism, is dysregulated in tissues undergoing chronic inflammation.

## 6. Conclusions

Chromatin modifiers are machines of pro- and anti- inflammatory responses that respond to pathological demands. Chromatin modifications are also essential in the regulation of metabolism. However, nucleosomes become less rigidly structured during aging, and histone levels are also reduced. Therefore, the restoration of homeostasis following exposure to noxious stimuli may become less responsive in aged cells. This being the case, the reduction of risk factors, such as dietary control, is shown to be of great importance to reduce inflammaging and age-related metabolic disorders. In parallel, the reduced transcription rate during aging may reduce the risk of the amplification of mutations, which can be caused by noxious stimuli. Further mechanistic studies of chromatin regulation with aging will advance our understanding of the balance between inflammaging and metabolism.
